# Associations between Serum Interleukins (IL-1*β*, IL-2, IL-4, IL-6, IL-8, and IL-10) and Disease Severity of COVID-19: A Systematic Review and Meta-Analysis

**DOI:** 10.1155/2022/2755246

**Published:** 2022-04-30

**Authors:** Yuanmin Chang, Mengru Bai, Qinghai You

**Affiliations:** Department of Respiratory and Critical Care Medicine, The First Affiliated Hospital of Anhui Medical University, China

## Abstract

**Background:**

To investigate the association between interleukins (IL-1*β*, IL-2, IL-4, IL-6, IL-8, and IL-10) and the disease severity of coronavirus disease 2019 (COVID-19).

**Materials and Methods:**

We systematically searched records investigating the role of interleukins (IL-1*β*, IL-2, IL-4, IL-6, IL-8, and IL-10) in COVID-19 patients in Web of Science, Pubmed, and Embase through December 2020. Data were extracted and pooled, and the weighted mean difference (WMD) and its 95% confidence interval (CI) were calculated. The funnel plot and the nonparametric trim and fill method were used to visualize and adjust the publication bias.

**Results:**

In total, 61 studies enrolled 14,136 subjects (14,041 patients and 95 healthy subjects) were enrolled in this meta-analysis. Our results showed that serum IL-2, IL-4, IL-6, and IL-10 levels were elevated in COVID-19 patients compared to healthy controls, and IL-6, IL-8, and IL-10 levels were increased in severe COVID-19 cases compared to nonsevere patients. Additionally, the levels of IL-1*β*, IL-6, and IL-8 were elevated in nonsurvivor patients compared to survivors. For patients in the intensive care unit (ICU), IL-6 and IL-8 levels were increased than that in non-ICU patients.

**Conclusions:**

Elevated levels of IL-6, IL-8, and IL-10 were associated with the disease severity of COVID-19, and elevated levels of IL-1*β*, IL-6, and IL-8 were related to the prognosis of COVID-19 patients, which could be used to evaluate COVID-19 patients' disease severity and prognosis.

## 1. Introduction

The severe acute respiratory syndrome coronavirus 2 (SARS-CoV-2) occurred and spread rapidly in Wuhan, China, in 2019, which has received wide attention [1]. As the spread of SARS-CoV-2, the confirmed coronavirus disease 2019 (COVID-19) cases increased dramatically, leading to a public health emergency [2–5]. Fever, dry cough, and muscle aches were common symptoms of COVID-19 cases, and the manifestations varied greatly in critically ill COVID-19 patients [6, 7]. With the development of the detection method, the COVID-19 cases could be confirmed timely to achieve early diagnosis and treatment [8]. Although therapeutic strategies for COVID-19 have advanced greatly, including antiviral drugs, vaccines, and immunomodulatory agents [9], older COVID-19 patients tend to develop severe disease status [10]. Hence, more effective treatment approaches for COVID-19 were warranted.

Immune responses were demonstrated to be involved in the initiation and development of COVID-19, and cytokine storm may cause a poor prognosis in COVID-19 patients [11–13]. Mehta et al. proposed that cytokine storm syndrome may be associated with the disease severity of COVID-19 patients, and immunosuppression could be a therapy option for COVID-19 patients [11]. Increasing evidence demonstrated that interleukins (ILs) played an important role in the progression of COVID-19. Compared to mild COVID-19 cases, serum interleukins levels increased greatly in severe and critical patients [13–17]. Additionally, the cytokine profiles were different between survivors and nonsurvivors of COVID-19 patients [18]. IL-6 level was reported to be associated with patients' clinical manifestations, including body temperature and blood oxygen saturation, and COVID-19 patients with higher IL-6 levels had a poorer prognosis [19]. Therapeutic agents targeting IL-6 have been applied to clinical practice, which improved the outcomes of severe and critical COVID-19 patients [20, 21]. Thus, immunomodulatory agents targeting immune mediators provided novel clues for the treatment of COVID-19.

In this meta-analysis, we comprehensively analyzed the levels of serum interleukins in COVID-19 patients according to disease severity. Our results showed that elevated levels of IL-6, IL-8, and IL-10 were associated with the disease severity of COVID-19, and elevated levels of IL-1*β*, IL-6, and IL-8 were associated with the prognosis of COVID-19 cases, and more studies were needed to elucidate the roles of interleukins in the progression and prognosis of COVID-19 to improve the outcomes of patients.

## 2. Materials and Methods

### 2.1. Search Study

All procedures in this study were performed according to the Preferred Reporting Items for Systematic Reviews and Meta-Analyses (PRISMA) statement [22]. Records in Web of Science, Pubmed, and Embase were searched up to December 15, 2020. We used the following search strategy: (“novel coronavirus” OR “SARS-CoV-2” OR “2019-nCoV” OR “COVID-19” OR “coronavirus disease 2019”) AND (“IL-1” OR “interleukin-1” OR “IL-2” OR “interleukin-2” OR “IL-4” OR “interleukin-4” OR “IL-6” OR “interleukin-6” OR “IL-8” OR “interleukin-8” OR “IL-10” OR “interleukin-10”).

Two investigators (Y.M.C.) and (M.R.B.) researched all relevant articles, and articles that fulfilled the inclusion criteria were included. Any disagreement would be discussed until an agreement was reached. The disease severity of COVID-19 was already defined in the included studies based on clinical criteria, which was according to the Novel Coronavirus Pneumonia Diagnosis and Treatment Plan, the Novel Coronavirus Pneumonia Diagnosis and Treatment Guidance, and World Health Organization (WHO) guidance [23–26], and COVID-19 patients with acute respiratory distress syndrome (ARDS) was defined as severe disease.

### 2.2. Study Selection and Data Extraction

The inclusion and exclusion criteria were used to identify relevant articles. Case reports, commentaries, meta-analyses, letters, reviews, animal trials, and editorials were excluded. The inclusion criteria were listed below: (i) patients with COVID-19 were confirmed by laboratory test; (ii) subgroup analysis was conducted according to disease severity; (iii) serum interleukin levels (IL-1*β*, IL-2, IL-4, IL-6, IL-8, and IL-10) were detected, which were expressed as median (q1-q3) or mean ± standard deviation (SD). The exclusion criteria were as follows: (i) patients were not diagnosed as COVID-19 cases or pediatric and pregnant COVID-19 cases; (ii) subgroup analysis was not conducted according to patient disease severity; (iii) data were not expressed as median (q1-q3) or mean ± SD, or data could not be transformed into mean ± SD; (iv) relevant serum interleukin (IL-1*β*, IL-2, IL-4, IL-6, IL-8, and IL-10) levels were not detected; and (v) repetitive publication. Two reviewers (Y.M.C) and (M.R.B) extracted the data from the selected studies, and the following items were extracted: first author, publication, country, number of subjects, median age, time of blood sampling, mean, SD, median, or interquartile of interleukins levels. The Newcastle-Ottawa Scale (NOS) tool was used to evaluate the quality of the included studies [27].

### 2.3. Statistical Analysis

All procedures were conducted in the R software. For data presented as median (q1-q3), the formulas mean = (q1 + m + q3)/3 and SD = (q3 − q1)/1.35 were used to transform the data into mean ± SD [28]. The weighted mean difference (WMD) and corresponding 95% confidence interval (CI) were calculated to compare the difference in serum interleukin levels between the two groups. The heterogeneity was assessed by *I*^2^, and a fixed-effects model was applied when *I*^2^ < 50%; otherwise, a random-effects model was adopted. The funnel plot and nonparametric trim and fill method were used to visualize and adjust the publication bias [29]. *P* < 0.05 (two sides) was recognized as significant difference.

## 3. Results

### 3.1. Basic Information of Included Studies

**Figure 1:**
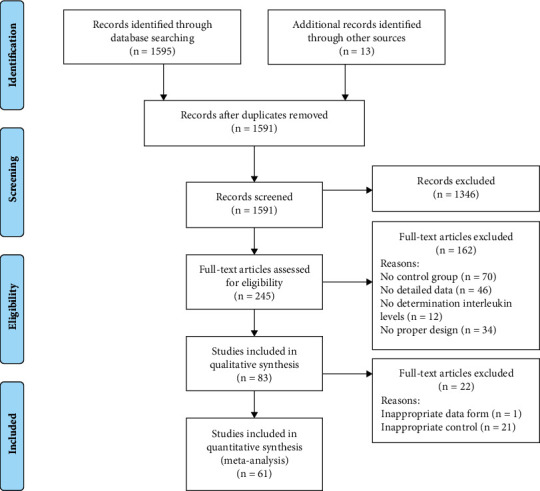
The Preferred Reporting Items for Systematic Reviews and Meta-Analyses flow chart.

Initially, 1591 articles were searched. After reviewing the titles and abstracts, 245 records were included, and 1346 records were excluded. Finally, after reviewing the full length, 61 studies including 14,136 subjects (14,041 patients and 95 healthy individuals), were integrated into our meta-analysis. [18, 30–89]. The PRISMA chart and checklist showed the whole process of our meta-analysis (Figure 1 and Supplemental Table 1). Data in the 61 studies were presented in Supplemental Tables 2–7, and the NOS scores of the included 61 studies were shown in Supplemental Table 8.

### 3.2. Alterations of IL-1*β*, IL-2, and IL-4 in COVID-19 Patients

**Figure 2:**
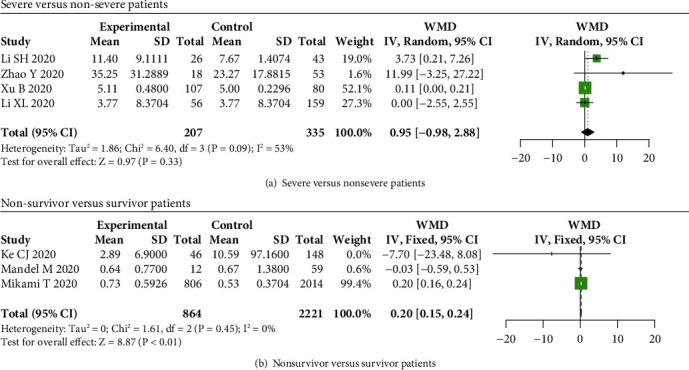
IL-1*β* levels in COVID-19 patients. A significant difference in IL-1*β* levels between severe and nonsevere COVID-19 patients was not found (*P* = 0.33) (a), while IL-1*β* levels were increased in (b) nonsurvivor patients compared to survivors.

To comprehensively elucidate the relationship between serum interleukins and disease severity of COVID-19 cases, we compared serum interleukin levels in COVID-19 patients with different disease severities. Our results showed that serum IL-1*β* levels were not elevated in severe COVID-19 patients compared to nonsevere patients (*P* = 0.33) (Figure 2(a)), while levels of IL-1*β* were elevated in nonsurvivor COVID-19 patients compared to survivors (WMD = 0.20, 95% CI: 0.15-0.24, and *P* < 0.01) (Figure 2(b)).

**Figure 3:**
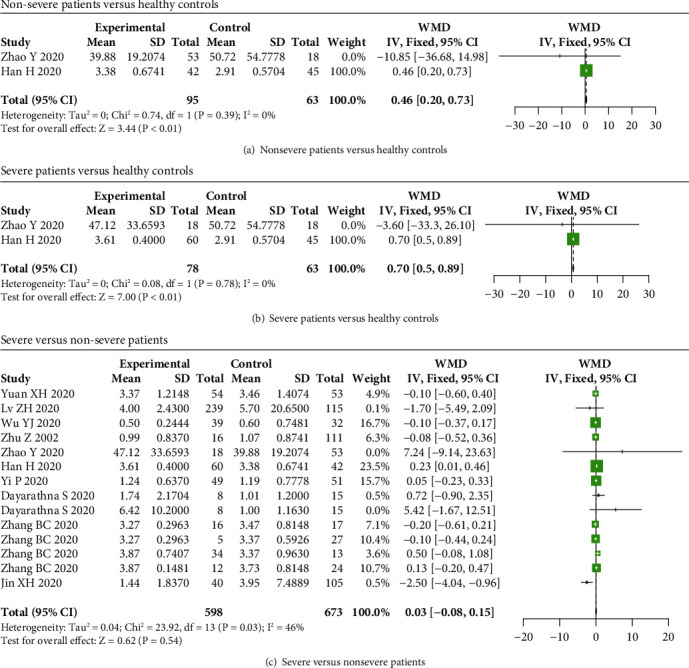
IL-2 levels in COVID-19 patients. The levels of IL-2 were increased in (a) nonsevere and (b) severe COVID-19 patients compared to healthy subjects, while no significant difference in IL-2 levels between (c) severe and nonsevere COVID-19 patients.

For IL-2 in COVID-19 patients, increased serum IL-2 levels were observed in nonsevere and severe patients than that in healthy controls (WMD = 0.46, 95% CI: 0.20-0.73, and *P* < 0.01; WMD = 0.70, 95% CI: 0.50-0.89, and *P* < 0.01) (Figures 3(a) and 3(b)), while no significant difference in IL-2 levels between severe and nonsevere patients (*P* = 0.54) (Figure 3(c)).

**Figure 4:**
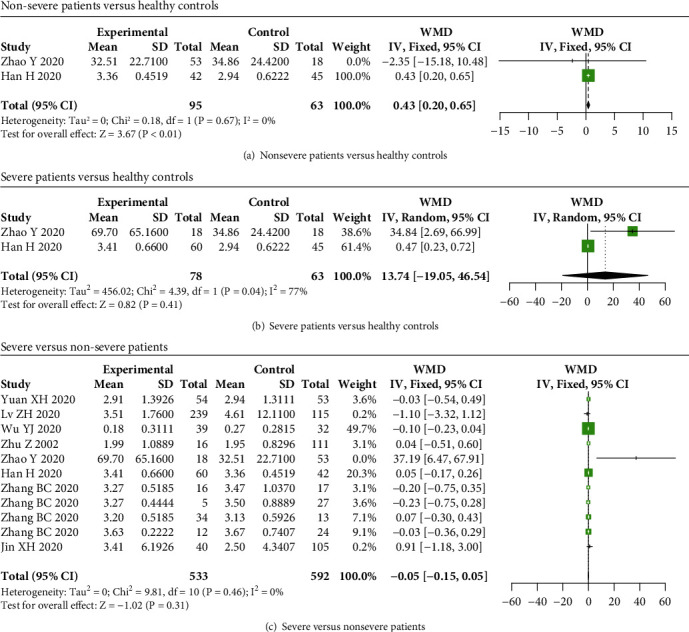
IL-4 levels in COVID-19 patients. IL-4 levels were elevated in (a) nonsevere COVID-19 patients compared to healthy subjects, while no significant difference in IL-4 levels between (b) severe patients and healthy controls, as well as between (c) severe and nonsevere COVID-19 patients.

Serum IL-4 levels were elevated in nonsevere COVID-19 patients compared to healthy individuals (WMD = 0.43, 95% CI: 0.20-0.65, and *P* < 0.01) (Figure 4(a)), while no significant difference in IL-4 levels was observed between healthy controls and severe patients, as well as between severe and nonsevere COVID-19 patients (*P* > 0.05) (Figures 4(b) and 4(c)).

### 3.3. Alterations of IL-6, IL-8, and IL-10 in COVID-19 Patients

**Figure 5:**
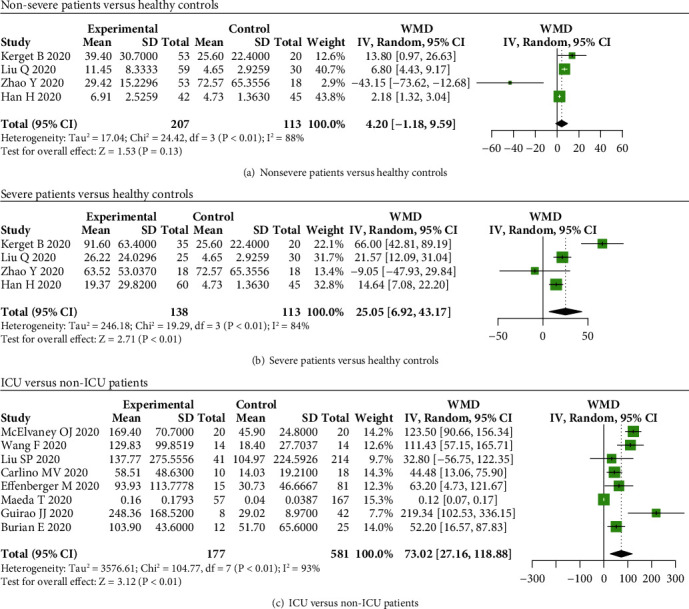
IL-6 levels in COVID-19 patients and healthy controls. No significant difference in serum IL-6 levels between (a) nonsevere COVID-19 patients and healthy controls, while IL-6 levels were elevated in (b) severe patients compared to healthy subjects, and levels of IL-6 were increased in (c) ICU patients compared to non-ICU patients.

**Figure 6:**
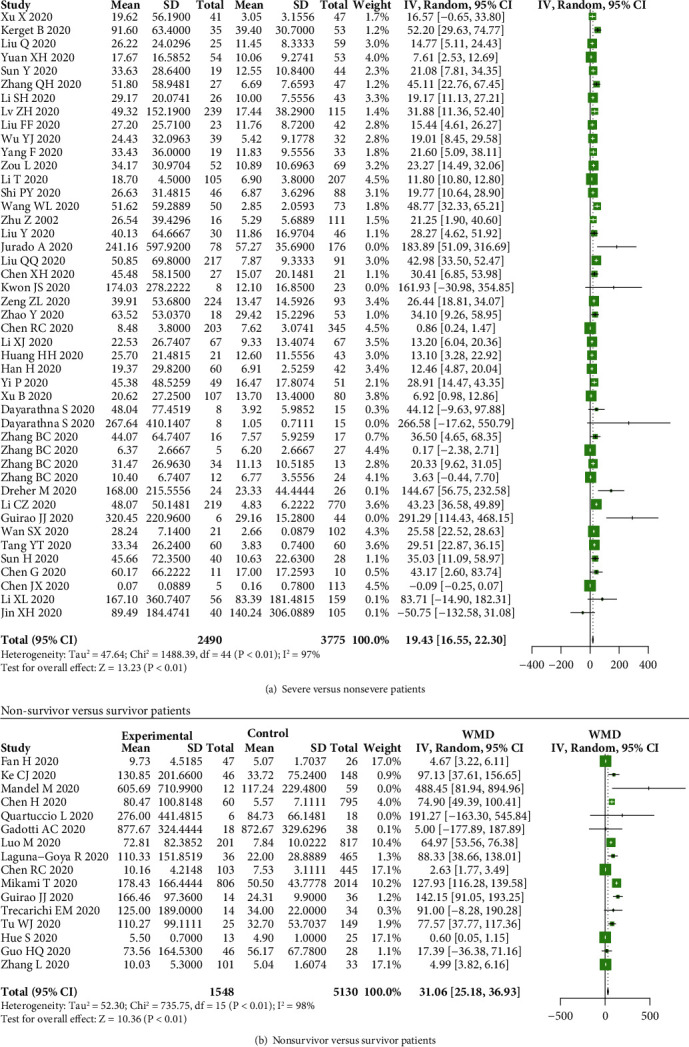
IL-6 levels in COVID-19 patients. The serum levels of IL-6 were increased in (a) severe and (b) nonsurvivor patients compared to nonsevere and survivor patients.

Our results indicated that serum IL-6 levels were not elevated in nonsevere COVID-19 cases compared to healthy controls (*P* = 0.13) (Figure 5(a)), while IL-6 levels were elevated in severe patients compared to healthy controls (WMD = 25.05, 95% CI: 6.92-43.17, and *P* < 0.01) (Figure 5(b)). We also found that the levels of IL-6 were elevated in intensive care unit (ICU), severe, and nonsurvivor patients than that in non-ICU, nonsevere, and survivor patients (WMD = 73.02, 95% CI: 27.16-118.88, and *P* < 0.01; WMD = 19.43, 95% CI: 16.55-22.30, and *P* < 0.01; WMD = 31.06, 95% CI: 25.18-36.93, and *P* < 0.01) (Figures 5(c) and 6(a) and 6(b)).

**Figure 7:**
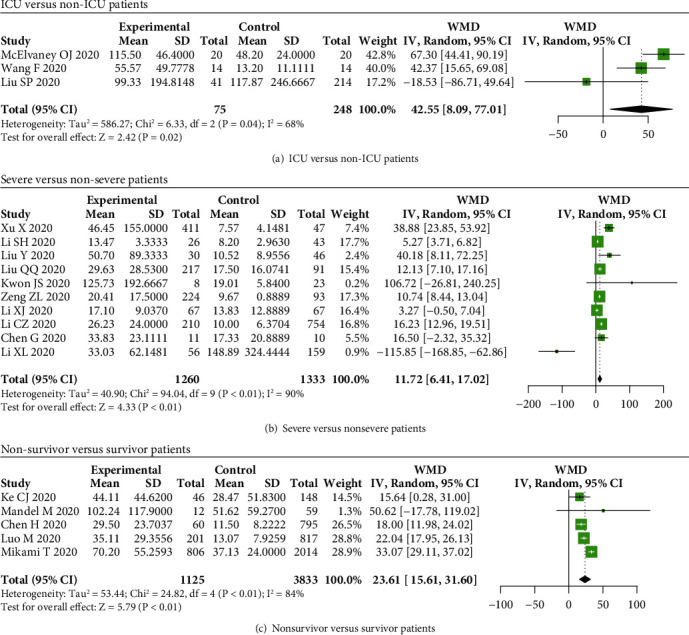
IL-8 levels in COVID-19 patients. The levels of IL-8 were increased in (a) ICU, (b) severe, and (c) nonsurvivor COVID-19 patients compared to non-ICU, nonsevere, and survivor patients.

The serum IL-8 levels were elevated in ICU, severe, and nonsurvivor COVID-19 patients compared to non-ICU, nonsevere, and survivor patients (WMD = 42.55, 95% CI: 8.09-77.01, and *P* = 0.02; WMD = 11.72, 95% CI: 6.41-17.02, and *P* < 0.01; WMD = 23.61, 95% CI: 15.61-31.60, and *P* < 0.01) (Figures 7(a)–7(c)).

**Figure 8:**
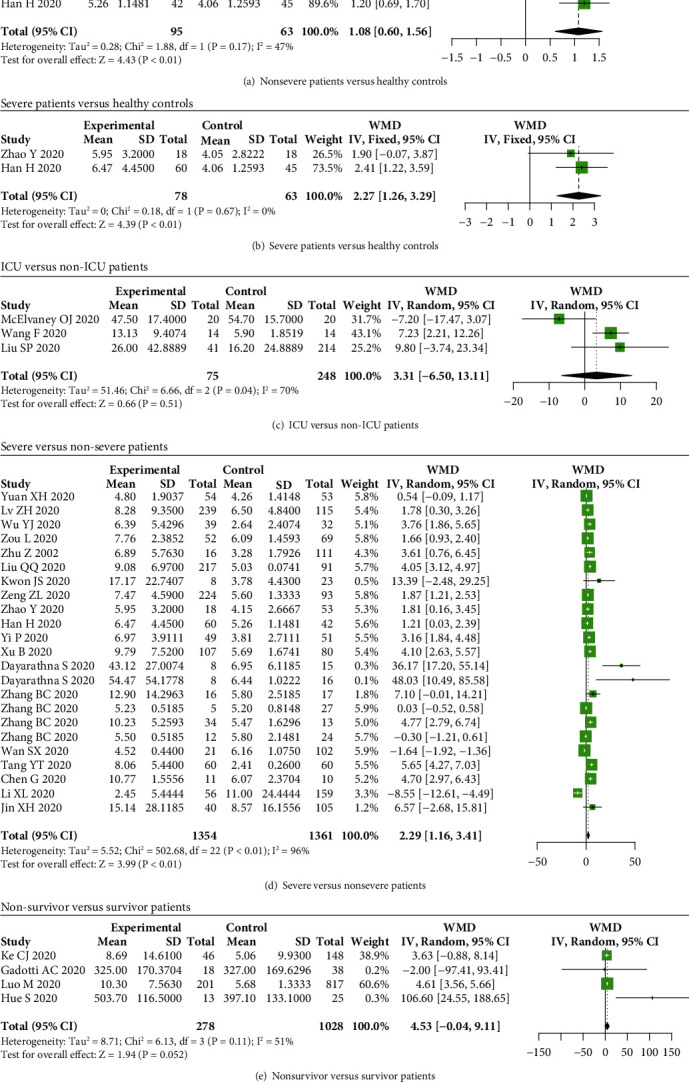
IL-10 levels in COVID-19 patients. The levels of IL-10 were elevated in (a) nonsevere and (b) severe patients compared to healthy controls, while no significant difference between (c) ICU and non-ICU patients, as well as between (e) nonsurvivor and survivor patients. The levels of IL-10 were elevated in (d) severe patients compared to nonsevere patients.

For IL-10, we found that serum IL-10 levels were increased in nonsevere and severe cases with COVID-19 compared to healthy subjects (WMD = 1.08, 95% CI: 0.60-1.56, and *P* < 0.01; WMD = 2.27, 95% CI: 1.26-3.29, and *P* < 0.01) (Figures 8(a) and 8(b)). However, IL-10 levels were not elevated between ICU and non-ICU patients, as well as between nonsurvivor and survivor patients (*P* > 0.05) (Figures 8(c) and 8(e)). Additionally, the serum IL-10 levels were higher in severe patients compared to nonsevere patients (WMD = 2.29, 95% CI: 1.16-3.41, and *P* < 0.01) (Figure 8(d)).

### 3.4. Publication Bias

In our meta-analysis, the potential publication bias was assessed and adjusted by funnel plot and the nonparametric trim and fill method, and Supplemental Figure 1 showed the publication bias for our meta-analysis, which was adjusted by the nonparametric trim and fill method.

## 4. Discussion

In this meta-analysis, we analyzed serum levels of IL-1*β*, IL-2, IL-4, IL-6, IL-8, and IL-10 in COVID-19 patients with different disease severities. Our main findings were as follows: (i) levels of IL-2, IL-4, IL-6, and IL-10 were elevated in COVID-19 patients compared to healthy subjects; (ii) levels of IL-6, IL-8, and IL-10 were elevated in severe COVID-19 cases compared to nonsevere patients, while no significant difference in IL-1*β*, IL-2, and IL-4 levels between severe and nonsevere patients; (iii) elevated levels of IL-1*β*, IL-6, and IL-8 were found in nonsurvivor COVID-19 patients compared to survivor ones; (iv) levels of IL-6 and IL-8 were elevated in ICU patients compared to non-ICU patients. Taken together, levels of IL-6, IL-8, and IL-10 were associated with the disease severity of COVID-19, and levels of IL-1*β*, IL-6, and IL-8 were correlated with the prognosis of COVID-19 patients, which may be used to predict the disease severity of COVID-19.

Since the outbreak of COVID-19, an increasing number of COVID-19 cases were confirmed. The cytokine storm occurred in COVID-19, and interleukins and IFN-*γ* were involved in the process of hyperinflammation [90]. Immune mediators including interleukins were demonstrated to play an important role in the development of COVID-19 [17, 91]. Tocilizumab, a kind of antibody that targeted the IL-6 signaling pathway, was demonstrated to be effective in treating severe COVID-19 patients, and biomarkers including C-reactive protein, procalcitonin, D-dimer, and lymphocyte levels were decreased after receiving tocilizumab administration [20, 92]. Hence, deepening the understanding of interleukins in the development of COVID-19 may contribute to its diagnosis and treatment.

In this study, we systematically analyzed the serum levels of IL-1*β*, IL-2, IL-4, IL-6, IL-8, and IL-10 in COVID-19 patients with different disease severities, as well as in healthy controls. Our results indicated that serum levels of IL-2, IL-4, IL-6, and IL-10 were increased in COVID-19 patients compared to healthy subjects. Additionally, we compared the levels of interleukins in severe and nonsevere COVID-19 patients, and the results indicated that the levels of IL-6, IL-8, and IL-10 were elevated in severe COVID-19 patients compared to nonsevere patients, while no significant difference in IL-1*β*, IL-2, and IL-4 levels between severe and nonsevere COVID-19 patients, implying that IL-6, IL-8, and IL-10 might be related to the disease severity of COVID-19. Then, we analyzed IL-1*β*, IL-6, IL-8, and IL-10 levels in nonsurvivor and survivor COVID-19 patients, and our results suggested that the levels of IL-1*β*, IL-6, and IL-8 levels were elevated in nonsurvivor patients compared to survivor patients, which indicated that IL-1*β*, IL-6, and IL-8 might be related to COVID-19 patients' prognosis. Compared to non-ICU patients, IL-6 and IL-8 levels were increased in ICU patients, which further demonstrated the important role of IL-6 and IL-8 in the pathogenesis of SARS-CoV-2 [93]. Taken together, these results showed that IL-6 and IL-8 were associated with the disease severity of COVID-19 patients, which may be used to predict patients' prognoses.

In conclusion, we found that serum levels of IL-6, IL-8, and IL-10 were associated with the disease severity of COVID-19 patients, and serum levels of IL-1*β*, IL-6, and IL-8 were associated with the prognosis of COVID-19 patients. Herein, more studies were needed to explore the immunological alterations underlying COVID-19 to improve its diagnosis and treatment.

The advantage of the meta-analysis was that more studies and more COVID-19 cases were included in our study, and we compared the levels of interleukins between COVID-19 cases and healthy subjects. Additionally, the COVID-19 patients included in our meta-analysis were from many countries, which makes the results to be more applicable worldwide. The limitation lies in that we used a random-effects model when great heterogeneity exists between studies, and pediatric and pregnant COVID-19 patients are excluded in our analysis, and our results may be not applied to them. Additionally, in our meta-analysis, the alterations of serum interleukin (IL-1*β*, IL-2, IL-4, IL-6, IL-8, and IL-10) levels in COVID-19 patients and healthy subjects were analyzed according to the following groups: (i) COVID-19 patients *vs*. healthy subjects, (ii) severe *vs*. nonsevere patients, (iii) survivor *vs*. nonsurvivor patients, and (iv) ICU *vs*. non-ICU patients. Because no relevant data was reported for the interleukins in some groups, not every kind of interleukin was analyzed according to the four groups, which caused the inconsistency in our results. In the next step, we will further comprehensively analyze the role of interleukins in the development of COVID-19.

## Data Availability

The data in the current study are available from the corresponding author on reasonable request.

## Supplementary Materials

Supplementary 1 Supplemental Figure 1: the funnel plots concerning IL-2 (A), IL-4 (B), IL-6 (C–E), IL-8 (F, G), and IL-10 (H) in our meta-analysis, and the publication biases were adjusted by the nonparametric trim and fill method.

Supplementary 2 Supplemental Table 1: the Preferred Reporting Items for Systematic Reviews and Meta-Analyses checklist. Supplemental Table 2: data extracted from enrolled studies concerning IL-1*β* in COVID-19 patients. Supplemental Table 3: data extracted from enrolled studies concerning IL-2 in COVID-19 patients and healthy controls. Supplemental Table 4: data extracted from enrolled studies concerning IL-4 in COVID-19 patients and healthy controls. Supplemental Table 5: data extracted from enrolled studies concerning IL-6 in COVID-19 patients and healthy controls. Supplemental Table 6: data extracted from enrolled studies concerning IL-8 in COVID-19 patients. Supplemental Table 7: data extracted from enrolled studies concerning IL-10 in COVID-19 patients and healthy controls. Supplemental Table 8: the Newcastle-Ottawa Scale (NOS) score showed the qualities of included studies.
