# Supplementary material for: Associations between Serum Interleukins (IL-1β, IL-2, IL-4, IL-6, IL-8, and IL-10) and Disease Severity of COVID-19: A Systematic Review and Meta-Analysis
Source: Biomed Res Int. 2022 Apr 30;2022:2755246. doi: 10.1155/2022/2755246 (PMC9079324; doi:10.1155/2022/2755246)
Supplement: Supplementary 1 — Supplemental Figure 1: the funnel plots concerning IL-2 (A), IL-4 (B), IL-6 (C–E), IL-8 (F, G), and IL-10 (H) in our meta-analysis, and the publication biases were adjusted by the nonparametric trim and fill method. [file 2755246.f1.pdf]

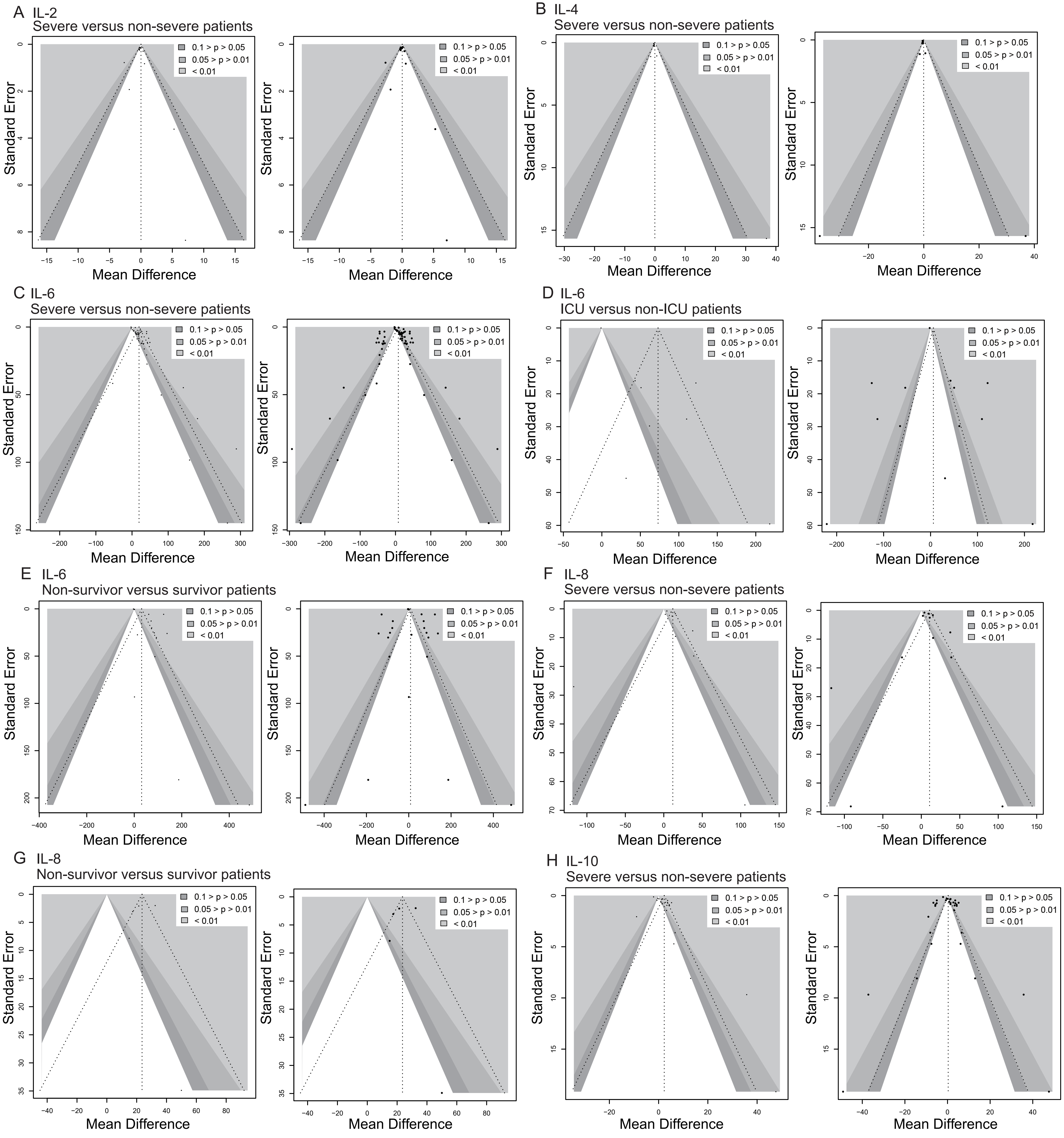
A IL-2
Severe versus non-severe patients
B IL-4
Severe versus non-severe patients
C IL-6
Severe versus non-severe patients
D IL-6
ICU versus non-ICU patients
E IL-6
Non-survivor versus survivor patients
F IL-8
Severe versus non-severe patients
G IL-8
Non-survivor versus survivor patients
H IL-10
Severe versus non-severe patients
0.1 > p > 0.05
0.05 > p > 0.01
< 0.01
Standard Error
Mean Difference
