# Supplementary material for: Associations between Serum Interleukins (IL-1β, IL-2, IL-4, IL-6, IL-8, and IL-10) and Disease Severity of COVID-19: A Systematic Review and Meta-Analysis
Source: Biomed Res Int. 2022 Apr 30;2022:2755246. doi: 10.1155/2022/2755246 (PMC9079324; doi:10.1155/2022/2755246)
Supplement: Supplementary 2 — Supplemental Table 1: the Preferred Reporting Items for Systematic Reviews and Meta-Analyses checklist. Supplemental Table 2: data extracted from enrolled studies concerning IL-1β in COVID-19 patients. Supplemental Table 3: data extracted from enrolled studies concerning IL-2 in COVID-19 patients and healthy controls. Supplemental Table 4: data extracted from enrolled studies concerning IL-4 in COVID-19 patients and healthy controls. Supplemental Table 5: data extracted from enrolled studies concerning IL-6 in COVID-19 patients and healthy controls. Supplemental Table 6: data extracted from enrolled studies concerning IL-8 in COVID-19 patients. Supplemental Table 7: data extracted from enrolled studies concerning IL-10 in COVID-19 patients and healthy controls. Supplemental Table 8: the Newcastle-Ottawa Scale (NOS) score showed the qualities of included studies. [file 2755246.f2.zip › Supplemental Table 2.docx]

**Supplemental Table 2.** Data extracted from enrolled studies concerning IL-1β in COVID-19 patients

| Author (year) | country | Age (median /mean) | Time of sampling |  | CIOVID-19 patients | | | | | | | | | Healthy control(HC) | unit |
| --- | --- | --- | --- | --- | --- | --- | --- | --- | --- | --- | --- | --- | --- | --- | --- |
|  |  |  | **On hospital admission** | **Regular/ general/ ordinary** | **Mild/Moderate** | **non-severe/non-critical** | **Severe** | **Critical** | **Severe + Critical** | **non-survivor/died/death** | **Survivor/alive/survival** | **non-ICU** | **ICU** |  |  |
|  |  |  |  | n, mean (SD) or median (IQR) | n, mean (SD) or median (IQR) | n, mean (SD) or median (IQR) | n, mean (SD) or median (IQR) | n, mean (SD) or median (IQR) | n, mean (SD) or median (IQR) | n, mean (SD) or median (IQR) | n, mean (SD) or median (IQR) | n, mean (SD) or median (IQR) | n, mean (SD) or median (IQR) | n, mean (SD) or median (IQR) | pg/ml |
| Li SH（2020） | China | - | On hospital admission |  |  | 43,7.5（6.8,8.7） | 26, 9.9 (6, 18.3) |  |  |  |  |  |  |  | pg/ml |
| Ke CJ（2020） | China | 62.55(14.18) | On hospital admission |  |  |  |  |  |  | 46, 2.89（6.9） | 148,10.59（97.16） |  |  |  | pg/ml |
| Mandel M（2020） | Israel | 62 (13.8) | On hospital admission |  |  |  |  |  |  | 12, 0.64（0.77） | 59, 0.67(1.38） |  |  |  | pg/ml |
| Chen H(2020) | China | 63 (52, 70) | On hospital admission |  |  |  |  |  |  | 60, 56.5(24.4, 160.5) | 795, 3.7 (1.7, 11.3) |  |  |  | pg/ml |
| Zhao Y(2020) | China | 48 (37, 63) | On hospital admission |  | 53, 22.58 (11.54, 35.68) (mild) |  | 18, 29.08 (17.22, 59.46) |  |  |  |  |  |  |  | pg/ml |
| Xu B（2020） | China | 62 (48.5, 71) | On hospital admission |  | 80, 4.9 (4.9, 5.21) (mild) |  | 45, 4.9 (4.9, 5.42) | 62, 4.9 (4.9, 5.61) |  |  |  |  |  |  | pg/ml |
| Mikami T(2020) | USA | 62（49, 73），76（65, 85） | On hospital admission |  |  |  |  |  |  | 806, 0.6(0.4, 1.2) | 2014, 0.5(0.3, 0.8) |  |  |  | pg/ml |
| Li XL(2020) | China | 44（32, 52），56.5（20, 72） | On hospital admission |  |  | 159, 0 (0, 11.3) | 56, 0 (0, 11.3) |  |  |  |  |  |  |  | pg/ml |

HC: healthy control.
