# Supplementary material for: Associations between Serum Interleukins (IL-1β, IL-2, IL-4, IL-6, IL-8, and IL-10) and Disease Severity of COVID-19: A Systematic Review and Meta-Analysis
Source: Biomed Res Int. 2022 Apr 30;2022:2755246. doi: 10.1155/2022/2755246 (PMC9079324; doi:10.1155/2022/2755246)
Supplement: Supplementary 2 — Supplemental Table 1: the Preferred Reporting Items for Systematic Reviews and Meta-Analyses checklist. Supplemental Table 2: data extracted from enrolled studies concerning IL-1β in COVID-19 patients. Supplemental Table 3: data extracted from enrolled studies concerning IL-2 in COVID-19 patients and healthy controls. Supplemental Table 4: data extracted from enrolled studies concerning IL-4 in COVID-19 patients and healthy controls. Supplemental Table 5: data extracted from enrolled studies concerning IL-6 in COVID-19 patients and healthy controls. Supplemental Table 6: data extracted from enrolled studies concerning IL-8 in COVID-19 patients. Supplemental Table 7: data extracted from enrolled studies concerning IL-10 in COVID-19 patients and healthy controls. Supplemental Table 8: the Newcastle-Ottawa Scale (NOS) score showed the qualities of included studies. [file 2755246.f2.zip › Supplemental Table 3.docx]

**Supplemental Table 3.** Data extracted from enrolled studies concerning IL-2 in COVID-19 patients and healthy controls.

| Author (year) | country | Age (median /mean) | Time of sampling |  | CIOVID-19 patients | | | | | | | | | Healthy control(HC) | unit |
| --- | --- | --- | --- | --- | --- | --- | --- | --- | --- | --- | --- | --- | --- | --- | --- |
|  |  |  | **On hospital admission** | **Regular/ general/ ordinary** | **Mild/Moderate** | **non-severe/non-critical** | **Severe** | **Critical** | **Severe + Critical** | **non-survivor/died/death** | **Survivor/alive/survival** | **non-ICU** | **ICU** |  |  |
|  |  |  |  | n, mean (SD) or median (IQR) | n, mean (SD) or median (IQR) | n, mean (SD) or median (IQR) | n, mean (SD) or median (IQR) | n, mean (SD) or median (IQR) | n, mean (SD) or median (IQR) | n, mean (SD) or median (IQR) | n, mean (SD) or median (IQR) | n, mean (SD) or median (IQR) | n, mean (SD) or median (IQR) | n, mean (SD) or median (IQR) | pg/ml |
| Yuan XH (2020) | China | 66 (52, 69)，68 (61, 76)，67.5 (57, 85) | On hospital admission | 53, 3.59 (2.45, 4.35) |  |  |  |  | 54, 3.52 (2.47, 4.11) |  |  |  |  |  | pg/ml |
| Lv ZH（2020） | China | 62 (23, 90) | On hospital admission | 115, 5.7 (20.65) |  |  | 155, 3.92 (2.25) | 84, 4.14 (2.74) |  |  |  |  |  |  | pg/ml |
| Wu YJ（2020） | China | 61 (49, 69) | On hospital admission |  | 32, 0.36 (0.22, 1.23) |  | 39, 0.52 (0.33, 0.66) |  |  |  |  |  |  |  | pg/ml |
| Zhu Z（2020） | China | 50.90(15.26) | On hospital admission |  |  | 111, 0.93 (0.55, 1.73) | 16, 0.9 (0.47, 1.6) |  |  |  |  |  |  |  | pg/ml |
| Zhao Y（2020） | China | 48 (37, 63)，48 (40.75, 52.25) | On hospital admission |  | 53, 36.76 (28.47, 54.4) |  | 18,45.96(24.98,70.42) |  |  |  |  |  |  | 18, 42.84 (17.69, 91.64) | pg/ml |
| Han H（2020） | China | -  59.8(9.7)（HC） | On hospital admission |  | 42, 3.4 (2.91, 3.82) |  | 43, 3.67 (3.45, 3.91) | 17, 3.32 (3.15, 3.81) |  |  |  |  |  | 45, 2.89 (2.54, 3.31) | pg/ml |
| Yi P（2020） | China | 54 (42, 64) | On hospital admission |  |  | 51, 0.95 (0.78, 1.83) | 49, 0.95 (0.95, 1.81) |  |  |  |  |  |  |  | pg/ml |
| Dayarathna S（2020） |  |  | day 4–9 of illness |  | 15, 0.8 (0.31, 1.93) |  | 8, 1.06 (0.61, 3.54) |  |  |  |  |  |  |  | pg/ml |
| Dayarathna S（2020） |  |  | day 10–21of illness |  | 15, 0.8 (0.31, 1.88) |  | 8, 3.08 (1.2, 14.97) |  |  |  |  |  |  |  | pg/ml |
| Zhang BC(2020) | China | 62(47,78),  62.5(54.0,69.0) | On hospital admission |  |  | 17, 3.3 (3,4.1) | 16, 3.2 (3.1,3.5) |  |  |  |  |  |  |  | pg/ml |
| Zhang BC(2020) | China | 49(37,58),  48.0 (36,57) | On hospital admission |  |  | 27, 3.3 (3,3.8) | 5, 3.2 (3.1,3.5) |  |  |  |  |  |  |  | pg/ml |
| Zhang BC(2020) | China | 66.5(56,73),  70.5(63.0,78.0) | On hospital admission |  |  | 13, 3.4 (2.7,4) | 34, 4 (3.3,4.3) |  |  |  |  |  |  |  | pg/ml |
| Zhang BC(2020) | China | 59.5(54.5,64),  62.0(52.0,74.5) | On hospital admission |  |  | 24, 3.9 (3.1,4.2) | 12, 3.8 (3.8,4) |  |  |  |  |  |  |  | pg/ml |
| Jin XH(2020) | China |  |  |  |  | 105, 1.35 (0.19,10.3) | 40, 1.19 (0.33,2.81) |  |  |  |  |  |  |  | pg/ml |

HC: healthy control.
