# Supplementary material for: Associations between Serum Interleukins (IL-1β, IL-2, IL-4, IL-6, IL-8, and IL-10) and Disease Severity of COVID-19: A Systematic Review and Meta-Analysis
Source: Biomed Res Int. 2022 Apr 30;2022:2755246. doi: 10.1155/2022/2755246 (PMC9079324; doi:10.1155/2022/2755246)
Supplement: Supplementary 2 — Supplemental Table 1: the Preferred Reporting Items for Systematic Reviews and Meta-Analyses checklist. Supplemental Table 2: data extracted from enrolled studies concerning IL-1β in COVID-19 patients. Supplemental Table 3: data extracted from enrolled studies concerning IL-2 in COVID-19 patients and healthy controls. Supplemental Table 4: data extracted from enrolled studies concerning IL-4 in COVID-19 patients and healthy controls. Supplemental Table 5: data extracted from enrolled studies concerning IL-6 in COVID-19 patients and healthy controls. Supplemental Table 6: data extracted from enrolled studies concerning IL-8 in COVID-19 patients. Supplemental Table 7: data extracted from enrolled studies concerning IL-10 in COVID-19 patients and healthy controls. Supplemental Table 8: the Newcastle-Ottawa Scale (NOS) score showed the qualities of included studies. [file 2755246.f2.zip › Supplemental Table 4.docx]

**Supplemental Table 4.** Data extracted from enrolled studies concerning IL-4 in COVID-19 patients and healthy controls.

| Author (year) | country | Age (median /mean) | Time of sampling |  | CIOVID-19 patients | | | | | | | | | Healthy control(HC) | unit |
| --- | --- | --- | --- | --- | --- | --- | --- | --- | --- | --- | --- | --- | --- | --- | --- |
|  |  |  | **On hospital admission** | **Regular/ general/ ordinary** | **Mild/Moderate** | **non-severe/non-critical** | **Severe** | **Critical** | **Severe + Critical** | **non-survivor/died/death** | **Survivor/alive/survival** | **non-ICU** | **ICU** |  |  |
|  |  |  |  | n, mean (SD) or median (IQR) | n, mean (SD) or median (IQR) | n, mean (SD) or median (IQR) | n, mean (SD) or median (IQR) | n, mean (SD) or median (IQR) | n, mean (SD) or median (IQR) | n, mean (SD) or median (IQR) | n, mean (SD) or median (IQR) | n, mean (SD) or median (IQR) | n, mean (SD) or median (IQR) | n, mean (SD) or median (IQR) | pg/ml |
| Yuan XH (2020) | China | 66 (52, 69)，68 (61, 76)，67.5(57,85) | On hospital admission | 53, 2.89 (2.08, 3.85) |  |  |  |  | 54, 3 (1.93, 3.81) |  |  |  |  |  | pg/ml |
| Lv ZH（2020） | China | 62 (23,90) | On hospital admission |  | 115, 4.61 (12.11) |  | 155, 3.41 (1.12) | 84, 3.7 (2.54) |  |  |  |  |  |  | pg/ml |
| Wu YJ（2020） | China | 61 (49,69) | On hospital admission |  | 32, 0.24 (0.1, 0.48) |  | 39, 0.11 (0, 0.42) |  |  |  |  |  |  |  | pg/ml |
| Zhu Z（2020） | China | 50.90(15.26) | On hospital admission |  |  | 111, 1.87 (1.43, 2.55) | 16, 1.99(1.26, 2.73) |  |  |  |  |  |  |  | pg/ml |
| Zhao Y（2020） | China | 48 (37,63) | On hospital admission |  | 53, 32.51 (22.71) |  | 18, 69.7 (65.16) |  |  |  |  |  |  | 18, 34.86 (24.42) | pg/ml |
| Han H（2020） | China | -  59.8(9.7)（HC） | On hospital admission |  | 42, 3.36 (3.06, 3.67) |  | 43, 3.44 (2.99, 3.92) | 17, 3.27 (2.92, 3.71) |  |  |  |  |  | 45, 2.79 (2.59, 3.43) | pg/ml |
| Zhang BC（2020） | China | 62(47,78),  62.5(54.0,69.0) | On hospital admission |  |  | 17, 3.2 (2.9, 4.3) | 16, 3.3 (2.9, 3.6) |  |  |  |  |  |  |  | pg/ml |
| Zhang BC（2020） | China | 49(37,58),  48.0 (36,57) | On hospital admission |  |  | 27, 3.3 (3, 4.2) | 5, 3.2 (3, 3.6) |  |  |  |  |  |  |  | pg/ml |
| Zhang BC（2020） | China | 66.5(56,73),  70.5(63.0,78.0) | On hospital admission |  |  | 13, 3 (2.8, 3.6) | 34, 3.1 (2.9, 3.6) |  |  |  |  |  |  |  | pg/ml |
| Zhang BC（2020） | China | 59.5(54.5,64),  62.0(52.0,74.5) | On hospital admission |  |  | 24, 3.6 (3.2, 4.2) | 12, 3.6 (3.5, 3.8) |  |  |  |  |  |  |  | pg/ml |
| Jin XH（2020） | China |  | On hospital admission |  |  | 105, 1.45 (0.1, 5.96) | 40, 1.54 (0.17, 8.53) |  |  |  |  |  |  |  | pg/ml |

HC: healthy control.
