# Supplementary material for: Associations between Serum Interleukins (IL-1β, IL-2, IL-4, IL-6, IL-8, and IL-10) and Disease Severity of COVID-19: A Systematic Review and Meta-Analysis
Source: Biomed Res Int. 2022 Apr 30;2022:2755246. doi: 10.1155/2022/2755246 (PMC9079324; doi:10.1155/2022/2755246)
Supplement: Supplementary 2 — Supplemental Table 1: the Preferred Reporting Items for Systematic Reviews and Meta-Analyses checklist. Supplemental Table 2: data extracted from enrolled studies concerning IL-1β in COVID-19 patients. Supplemental Table 3: data extracted from enrolled studies concerning IL-2 in COVID-19 patients and healthy controls. Supplemental Table 4: data extracted from enrolled studies concerning IL-4 in COVID-19 patients and healthy controls. Supplemental Table 5: data extracted from enrolled studies concerning IL-6 in COVID-19 patients and healthy controls. Supplemental Table 6: data extracted from enrolled studies concerning IL-8 in COVID-19 patients. Supplemental Table 7: data extracted from enrolled studies concerning IL-10 in COVID-19 patients and healthy controls. Supplemental Table 8: the Newcastle-Ottawa Scale (NOS) score showed the qualities of included studies. [file 2755246.f2.zip › Supplemental Table 5.docx]

**Supplemental Table 5.** Data extracted from enrolled studies concerning IL-6 in COVID-19 patients and healthy controls.

| Author (year) | country | Age (median /mean) | Time of sampling |  | CIOVID-19 patients | | | | | | | | | Healthy control(HC) | unit |
| --- | --- | --- | --- | --- | --- | --- | --- | --- | --- | --- | --- | --- | --- | --- | --- |
|  |  |  | **On hospital admission** | **Regular/ general/ ordinary** | **Mild/Moderate** | **non-severe/non-critical** | **Severe** | **Critical** | **Severe + Critical** | **non-survivor/died/death** | **Survivor/alive/survival** | **non-ICU** | **ICU** |  |  |
|  |  |  |  | n, mean (SD) or median (IQR) | n, mean (SD) or median (IQR) | n, mean (SD) or median (IQR) | n, mean (SD) or median (IQR) | n, mean (SD) or median (IQR) | n, mean (SD) or median (IQR) | n, mean (SD) or median (IQR) | n, mean (SD) or median (IQR) | n, mean (SD) or median (IQR) | n, mean (SD) or median (IQR) | n, mean (SD) or median (IQR) | pg/ml |
| Xu X (2020) | China | 57.11 | On hospital admission |  | 47, 1.88 (1.5, 5.76) |  | 32, 2.96 (1.89, 14.07) | 9, 34.01 (8.71, 158.1) |  |  |  |  |  |  | pg/ml |
| Kerget B（2020） | Atatürk | 49.1(21.1)（Patients）  35.2(6.9)（HC） | On hospital admission |  |  | 53, 39.4 (30.7) |  | 35, 91.6 (63.4) |  |  |  |  |  | 20, 25.6 (22.4) | pg/ml |
| Liu Q(2020) | China | 51 (37,59) | On hospital admission |  |  | 59, 11.56 (5.77, 17.02) | 25,20.5 (12.86, 45.3) |  |  |  |  |  |  | 30, 5.17(2.42, 6.37) | pg/ml |
| Fan H(2020) | China | 58.3（14.31) | On hospital admission |  |  |  |  |  |  | 47,9.1 (7, 13.1) | 26,4.9 (4, 6.3) |  |  |  | pg/ml |
| Yuan XH(2020) | China | 66 (52, 69)，68 (61, 76)，67.5 (57, 85) | On hospital admission | 53,7.75(4.96,17.48) |  |  |  |  | 54, 16.25 (7.19, 29.58) |  |  |  |  |  | pg/ml |
| Sun Y(2020) | China | 47（3,85） | On hospital admission |  | 8, 5.26 (1.25) (mild)  36,14.17 (11.37) （moderate） |  | 10, 33.22 (31.9) | 9, 34.09 (26.47) |  |  |  |  |  |  | pg/ml |
| McElvaney OJ(2020) | Ireland | 55.5 (17.7) | On hospital admission |  |  |  |  |  |  |  |  | 20, 45.9 (24.8) | 20, 169.4(70.7) |  | pg/ml |
| Zhang QH(2020) | China | 62(56, 72) | On hospital admission |  |  | 47,4.41 (2.66, 13.00) | 27,51.95(11.93,91.51) |  |  |  |  |  |  |  | pg/ml |
| Li SH(2020) | China |  | On hospital admission |  |  | 43,8.4 (5.7, 15.9) | 26,24.6 (17.9, 45) |  |  |  |  |  |  |  | pg/ml |
| Lv ZH(2020) | China | 62 (23,90) | On hospital admission |  | 115, 17.44 (38.29) |  | 155, 29.82 (82.47) | 84, 85.29 (227.55) |  |  |  |  |  |  | pg/ml |
| Liu FF(2020) | China | 48.4（18.46） | On hospital admission | 32, 13.76 (9.07) | 10, 5.36 (1.84) |  | 8, 15.94 (14.88) | 15, 33.21 (28.58) |  |  |  |  |  |  | pg/ml |
| Wu YJ(2020) | China | 61 (49,69) | On hospital admission |  | 32,2.21 (0.83, 13.22) |  | 39,18.15 (5.91, 49.24) |  |  |  |  |  |  |  | pg/ml |
| Yang F(2020) | China | 63（34,98） | On hospital admission |  | 33,10.2 (6.2, 19.1) |  |  |  | 19,33.7 (9, 57.6) |  |  |  |  |  | pg/ml |
| Zou L(2020) | China | 65.0(55.0,71.5) | On hospital admission |  |  | 69,7.7 (5.27, 19.71) | 52,28.91 (15.89, 57.7) |  |  |  |  |  |  |  | pg/ml |
| Wang F(2020) | China | 68.6 (9.0) | On hospital admission |  |  |  |  |  |  |  |  | 14,13 (2.4, 39.8) | 14,124.5 (65.1, 199.9) |  | pg/ml |
| Li T(2020) | China | 69.2(7.3) | On hospital admission |  |  | 207, 6.9(3.8) | 105, 18.7 (4.5) |  |  |  |  |  |  |  | pg/ml |
| Ke CJ(220) | China | 62.55(14.18) | On hospital admission |  |  |  |  |  |  | 46, 130.85(201.66) | 148, 33.72(75.24) |  |  |  | pg/ml |
| Shi PY(2020) | China | 46 (34,58) | On hospital admission |  |  | 88,5.7 (5, 9.9) | 46,23 (7.2, 49.7) |  |  |  |  |  |  |  | pg/ml |
| Wang WL(2020) | China | 68 (56.5,78.0) | On hospital admission |  |  | 73, 2.78 (1.5, 4.28) |  | 50,44.94 (14.94, 94.98) |  |  |  |  |  |  | pg/ml |
| Zhu Z(2020) | China | 50.90(15.26) | On hospital admission |  |  | 111,3.82 (2.19, 9.87) | 16,24.11 (1.14, 54.37) |  |  |  |  |  |  |  | pg/ml |
| Liu Y(2020) | China | 45 | On hospital admission |  | 46,7.1 (2.79, 25.7) |  | 30,29.1 (2, 89.3) |  |  |  |  |  |  |  | pg/ml |
| Jurado A(2020) | Spanish |  | On hospital admission |  | 78, 31.4 (40.53) (mild)  98,77.86(155.3)（moderate） |  | 78, 241.16 (597.92) |  |  |  |  |  |  |  | pg/ml |
| Mandel M(2020) | Israel | 62(13.8) | On hospital admission |  |  |  |  |  |  | 12, 605.69(710.99) | 59, 117.24(229.48) |  |  |  | pg/ml |
| Liu QQ(2020) | China | 55.0(39.0,67.0)，66.0 (55.5,73.0)，70.0 (63.3,78.8) | On hospital admission |  | 91,5.6 (2.7, 15.3) |  | 133,24.3 (6.7, 61.7) | 84,64.8 (29.42, 153.1) |  |  |  |  |  |  | pg/ml |
| Chen XH(2020) | China | 64.6(18.1) | On hospital admission |  | 21,10.4 (3.8, 31) |  | 10,5.8 (3.1, 16.9) | 17,64 (25.6, 111.9) |  |  |  |  |  |  | pg/ml |
| Chen H(2020) | China | 63 (52,70) | On hospital admission |  |  |  |  |  |  | 60,56.5 (24.4, 160.5) | 795,3.7 (1.7, 11.3) |  |  |  | pg/ml |
| Quartuccio L(2020) | Italy | 65.8(8.2)，  68.8( 9.4) | On hospital admission |  |  |  |  |  |  | 6,171 (30.5, 626.5) | 18,63.5 (50.7, 140) |  |  |  | pg/ml |
| Liu SP(2020) | China | 64 (24,92) | On hospital admission |  |  |  |  |  |  |  |  | 214,8.7 (1.5, 304.7) | 41,36.5 (2.4, 374.4) |  | pg/ml |
| Gadotti AC(2020) | Brazil | 61 (47,73) | On hospital admission |  |  |  |  |  |  | 18,889 (653, 1091) | 38,883 (645, 1090) |  |  |  | pg/ml |
| Luo M(2020) | China | 61(49,69) | On hospital admission |  |  |  |  |  |  | 201,56.16(25.53,136.75) | 817,5.36 (2.32, 15.85) |  |  |  | pg/ml |
| Laguna-Goya R(2020) | Spain | 52 (44,60) | On hospital admission |  |  |  |  |  |  | 36,86 (20, 225) | 465,17 (5, 44) |  |  |  | pg/ml |
| Zhao Y(20200 | China | 48(37,63)(Patients)  48(40.75,52.25)(HC) | On hospital admission |  | 53,25.18 (21.26, 41.82) |  | 18,55.73(31.62,103.22) |  |  |  |  |  |  | 18,77.78(25.85,114.08) | pg/ml |
| Chen RC(2020) | China | 56.0(14.5) | On hospital admission |  | 345,7.4(5.66, 9.81) |  | 155,8.43(6.18, 10.52) | 48,8.33(5.46, 12.63) |  | 103,9.74 (7.53, 13.22) | 445,7.24 (5.58, 9.78) |  |  |  | pg/ml |
| Carlino MV(2020) | Italy | 56 | On hospital admission |  |  |  |  |  |  |  |  | 18, 14.03(19.21) | 10, 58.51(48.63) |  | pg/ml |
| Han H(2020) | China | -  59.8(9.7)(HC) | On hospital admission |  | 42,6.23(5.55, 8.96) |  | 43,10.49(6.23, 20.91) | 17,19.48(11.28,79.14) |  |  |  |  |  | 45,4.79(3.78, 5.62) | pg/ml |
| Effenberger M(2020) | Austria | 60.69 (18.915) | On hospital admission |  |  |  |  |  |  |  |  | 81, 18.6(5.3, 68.3) | 15, 92.2(18, 171.6) |  | pg/ml |
| Maeda T(2020) | USA | 63(17) | On hospital admission |  |  |  |  |  |  |  |  | 167, 36.7(18.6, 70.8) | 57, 127(58.9, 301) |  | pg/L |
| Mikami T(2020) | USA | 62（49,73），  76（65,85） | On hospital admission |  |  |  |  |  |  | 806, 152.4(79.1, 303.8) | 2014, 45.8(23.3, 82.4) |  |  |  | pg/ml |
| Jin XH(2020) | China |  | On hospital admission |  |  | 105, 5.93(0.78,414) | 40, 13.9(2.76,251.8) |  |  |  |  |  |  |  | pg/ml |
| Li XL(2020) | China | 44 (32,52),  56.5 (20,72) | On hospital admission |  |  | 159, 5.18(0,245) | 56, 14.3(0,487) |  |  |  |  |  |  |  | pg/ml |
| Chen JX(2020) | China |  | On hospital admission | 92, 0.02(0.01,0.04) | 21, 0.01(0.01,2.32) |  |  |  | 5, 0.03(0.03,0.15) |  |  |  |  |  | pg/ml |
| Chen G(2020) | China | 56.0 (50.0,65.0) | On hospital admission |  | 10, 15.3(6.2,29.5) |  | 11, 41.5(24.8,114.2) |  |  |  |  |  |  |  | pg/ml |
| Sun H(2020) | China | 59.75(12.67),  52.94(11.11) | On hospital admission | 28, 10.63(22.63) |  |  | 40, 45.66(72.35) |  |  |  |  |  |  |  | pg/ml |
| Tang YT(2020) | China | 59 (47,68) | On hospital admission |  | 60, 3.83(0.74) |  | 28, 6.92 (1.29) | 32, 56.45 (11.26) |  |  |  |  |  |  | pg/ml |
| Wan SX(2020) | China | 43.05(13.12),  61.29(15.55) | On hospital admission |  | 102, 2.664 (0.08792) |  | 21, 28.24 (7.14) |  |  |  |  |  |  |  | pg/ml |
| Guirao JJ(2020) | Spain | 64.5(2.26),  65.47(2.05),  56.20(2.85) | On hospital admission |  | 10, 7.66 (4.52) (mild)  34,35.48(10.84)（moderate） |  | 6, 320.45(220.96) |  |  |  |  |  |  |  | pg/ml |
| Li CZ(2020) | China |  | On hospital admission |  |  | 770, 3.1(1.5,9.9) |  | 219, 40.1(18.2,85.9) |  |  |  |  |  |  | pg/ml |
| Dreher M(2020) | Germany | 65 (58,76) | On hospital admission |  |  | 26, 10(0,60) | 24, 119(47,338) |  |  |  |  |  |  |  | pg/ml |
| Zhang BC(2020) | China | 59.5 (54.5,64),  62.0 (52.0,74.5) | On hospital admission |  |  | 24, 6.9(4.3,9.1) | 12, 9.7(6.2,15.3) |  |  |  |  |  |  |  | pg/ml |
| Zhang BC(2020) | China | 66.5 (56,73),  70.5 (63.0,78.0) | On hospital admission |  |  | 13, 7.4(5.9,20.1) | 34, 24.8(16.6,53) |  |  |  |  |  |  |  | pg/ml |
| Zhang BC(2020) | China | 49 (37,58),  48.0 (36,57) | On hospital admission |  |  | 27, 6.2(4.4,8) | 5, 6.7(4.4,8) |  |  |  |  |  |  |  | pg/ml |
| Zhang BC(2020) | China | 62 (47,78),  62.5(54.0,69.0) | On hospital admission |  |  | 17, 5.7(4.5,12.5) | 16, 24.4(10.2,97.6) |  |  |  |  |  |  |  | pg/ml |
| Dayarathna S(2020) |  |  | day 10–21of illness |  | 15, 1.04(0.58,1.54) |  | 8,207.34(20.94,574.63) |  |  |  |  |  |  |  | pg/ml |
| Dayarathna S(2020) |  |  | day 4–9 of illness |  | 15, 2.06(0.81,8.89) |  | 8, 22.65(8.46,113.02) |  |  |  |  |  |  |  | pg/ml |
| Xu B(2020) | China | 62 (48.5,71) | On hospital admission |  | 80, 14.6(4.21,22.3) |  | 45, 11.3(4.34,28.43) | 62, 17.4(7.18,50.2) |  |  |  |  |  |  | pg/ml |
| Yi P(2020) | China | 54 (42,64) | On hospital admission |  |  | 51, 12.52(6.42,30.46) | 49, 38.22(16.2,81.71) |  |  |  |  |  |  |  | pg/ml |
| Huang HH(2020) | China | 47.8(18.5) | On hospital admission |  |  | 43, 10.6(5.8,21.4) | 21, 18.7(14.7,43.7) |  |  |  |  |  |  |  | pg/ml |
| Li XJ(2020) | China | 43 (38,47) | On hospital admission |  | 67, 6.5(1.7,19.8) |  | 67, 20.3(5.6,41.7) |  |  |  |  |  |  |  | pg/ml |
| Zeng ZL(2020) | China | 62.0 (51.0,70.0) | On hospital admission |  | 93, 13.1(3.8,23.5) |  | 167, 21.7(6.3,53.9) | 57, 59.7(33.5,137.4) |  |  |  |  |  |  | pg/ml |
| Kwon JS(2020) | Korea | 50 (3.3) | On hospital admission |  | 6, 5.1(2.7,10.1) (mild)  17,11.6(2.6,28.6)（moderate） |  |  |  | 8,68.3 (39.1, 414.7) |  |  |  |  |  | pg/ml |
| Guirao JJ(2020) | Spain | 62.13(2.81)，  63.76(1.80) | On hospital admission |  |  |  |  |  |  |  |  | 42, 29.02 (8.97) | 8, 248.36 (168.52) |  | pg/ml |
| Guirao JJ(2020) | Spain | 69.00(3.09)，  61.36(1.72) | On hospital admission |  |  |  |  |  |  | 14, 166.46(97.36) | 36, 24.31 (9.9) |  |  |  | pg/ml |
| Trecarichi EM(2020) | Italy | 85(8),  78(13) | On hospital admission |  |  |  |  |  |  | 14, 125 (189) | 34, 34 (22) |  |  |  | pg/ml |
| Tu WJ(2020) | China | 70 (64,80),  51 (37,62) | On hospital admission |  |  |  |  |  |  | 25, 108.8(44.1, 177.9) | 149, 16.8(4.4, 76.9) |  |  |  | pg/ml |
| Guo HQ(2020) | China | 67.2(14.6) | On hospital admission |  |  |  |  |  |  | 46, 73.56 (164.53) | 28, 56.17 (67.78) |  |  |  | pg/ml |
| Zhang L(2020) | China | 60.78(12.98) | On hospital admission |  |  |  |  |  |  | 101, 9.15 (6.90,14.05) | 33, 5.11 (3.92,6.09) |  |  |  | pg/ml |
| Burian E(2020) | Germany |  | On hospital admission |  |  |  |  |  |  |  |  | 25, 51.7 (65.6) | 12, 103.9 (43.6) |  | pg/ml |
| Hue S(2020) | France |  | On hospital admission |  |  |  |  |  |  | 13, 5.5 (0.7) | 25, 4.9 (1) |  |  |  | pg/ml |

HC: healthy control.
