# Supplementary material for: Associations between Serum Interleukins (IL-1β, IL-2, IL-4, IL-6, IL-8, and IL-10) and Disease Severity of COVID-19: A Systematic Review and Meta-Analysis
Source: Biomed Res Int. 2022 Apr 30;2022:2755246. doi: 10.1155/2022/2755246 (PMC9079324; doi:10.1155/2022/2755246)
Supplement: Supplementary 2 — Supplemental Table 1: the Preferred Reporting Items for Systematic Reviews and Meta-Analyses checklist. Supplemental Table 2: data extracted from enrolled studies concerning IL-1β in COVID-19 patients. Supplemental Table 3: data extracted from enrolled studies concerning IL-2 in COVID-19 patients and healthy controls. Supplemental Table 4: data extracted from enrolled studies concerning IL-4 in COVID-19 patients and healthy controls. Supplemental Table 5: data extracted from enrolled studies concerning IL-6 in COVID-19 patients and healthy controls. Supplemental Table 6: data extracted from enrolled studies concerning IL-8 in COVID-19 patients. Supplemental Table 7: data extracted from enrolled studies concerning IL-10 in COVID-19 patients and healthy controls. Supplemental Table 8: the Newcastle-Ottawa Scale (NOS) score showed the qualities of included studies. [file 2755246.f2.zip › Supplemental Table 6.docx]

**Supplemental Table 6.** Data extracted from enrolled studies concerning IL-8 in COVID-19 patients.

| Author (year) | country | Age (median /mean) | Time of sampling |  | CIOVID-19 patients | | | | | | | | | Healthy control(HC) | unit |
| --- | --- | --- | --- | --- | --- | --- | --- | --- | --- | --- | --- | --- | --- | --- | --- |
|  |  |  | **On hospital admission** | **Regular/ general/ ordinary** | **Mild/Moderate** | **non-severe/non-critical** | **Severe** | **Critical** | **Severe + Critical** | **non-survivor/died/death** | **Survivor/alive/survival** | **non-ICU** | **ICU** |  |  |
|  |  |  |  | n, mean (SD) or median (IQR) | n, mean (SD) or median (IQR) | n, mean (SD) or median (IQR) | n, mean (SD) or median (IQR) | n, mean (SD) or median (IQR) | n, mean (SD) or median (IQR) | n, mean (SD) or median (IQR) | n, mean (SD) or median (IQR) | n, mean (SD) or median (IQR) | n, mean (SD) or median (IQR) | n, mean (SD) or median (IQR) | pg/ml |
| Xu X (2020) | China | 57.11 | On hospital admission |  | 47, 7.1 (5, 10.6) |  | 32, 9.25 (7.25, 12.73) | 9, 76.1 (18.8, 436) |  |  |  |  |  |  | pg/ml |
| McElvaney OJ（2020） | Ireland | 55.5(17.7) | On hospital admission |  |  |  |  |  |  |  |  | 20, 48.2(24.0) | 20, 115.5 (46.4) |  | pg/ml |
| Li SH（2020） | China |  | On hospital admission |  |  | 43, 7.8 (6.4, 10.4) | 26, 13.1 (11.4, 15.9) |  |  |  |  |  |  |  | pg/ml |
| Wang F(2020) | China | 68.6( 9.0) | On hospital admission |  |  |  |  |  |  |  |  | 14, 11 (6.8, 21.8) | 14, 49.1 (25.2, 92.4) |  | pg/ml |
| Ke CJ(2020) | China | 62.55(14.18) | On hospital admission |  |  |  |  |  |  | 46, 44.11 (44.62) | 148, 28.47 (51.83) |  |  |  | pg/ml |
| Liu Y(2020) | China | 45 | On hospital admission |  | 46, 8.66 (5.41, 17.5) |  | 30, 21.5 (5, 125.6) |  |  |  |  |  |  |  | pg/ml |
| Mandel M(2020) | Israel | 62±13.8(Patients)  48.9 ± 8.4(HC) | On hospital admission |  |  |  |  |  |  | 12, 102.24 (117.9) | 59, 51.62 (59.27) |  |  |  | pg/ml |
| Liu QQ(2020) | China | 55.0 (39.0, 67.0)，66.0 (55.5, 73.0)，70.0 (63.3, 78.8) | On hospital admission |  | 91, 15.4 (7.7, 29.4) |  | 133, 19.5 (12, 35.5) | 84, 30.8 (21, 71.8) |  |  |  |  |  |  | pg/ml |
| Chen H(2020) | China | 63 (52, 70) | On hospital admission |  |  |  |  |  |  | 60, 25.1 (15.7, 47.7) | 795, 10.2 (6.6, 17.7) |  |  |  | pg/ml |
| Kwon JS(2020) | Korea | 50(3.3) | On hospital admission |  | 6, 14.8 (8.3, 20.7) (mild)  17,20.0(11.9,29.8) (moderate) |  |  |  | 8, 52.7 (32.2, 292.3) |  |  |  |  |  | pg/ml |
| Liu SP(2020) | China | 64 (24, 92) | On hospital admission |  |  |  |  |  |  |  |  | 214, 10.6 (5, 338) | 41, 25 (5, 268) |  | pg/ml |
| Luo M(2020) | China | 61(49, 69) | On hospital admission |  |  |  |  |  |  | 201, 29.9(17.9, 57.53) | 817, 12.31 (8.1, 18.8) |  |  |  | pg/ml |
| Zeng ZL(2020) | China | 62.0 (51.0, 70.0) | On hospital admission |  | 93, 12.2 (7.8, 9) |  | 167, 15.5 (9.7, 26.1) | 57, 26 (14.9, 49.4) |  |  |  |  |  |  | pg/ml |
| Li XJ(2020) | China | 43 (38, 47) | On hospital admission |  | 67, 10.3 (6.9, 24.3) |  | 67, 18.5 (10.3, 22.5) |  |  |  |  |  |  |  | pg/ml |
| Mikami T(2020) | USA | 62（49, 73），  76（65, 85） | On hospital admission |  |  |  |  |  |  | 806, 60.2(37.9, 112.5) | 2014, 34.6 (22.2, 54.6) |  |  |  | pg/ml |
| Li CZ(2020) | China |  | On hospital admission |  |  | 754, 9 (6.2, 14.8) |  | 210, 21.9 (12.2, 44.6) |  |  |  |  |  |  | pg/ml |
| Chen G(2020) | China | 56.0 (50.0, 65.0) | On hospital admission |  | 10, 11 (6.4, 34.6) |  | 11, 34.9 (17.7, 48.9) |  |  |  |  |  |  |  | pg/ml |
| Li XL(2020) | China | 44 (32, 52),  56.5 (20, 72) | On hospital admission |  |  | 159, 8.66 (0, 438) | 56, 15.2 (0, 83.9) |  |  |  |  |  |  |  | pg/ml |

HC: healthy control.
