# Supplementary material for: Associations between Serum Interleukins (IL-1β, IL-2, IL-4, IL-6, IL-8, and IL-10) and Disease Severity of COVID-19: A Systematic Review and Meta-Analysis
Source: Biomed Res Int. 2022 Apr 30;2022:2755246. doi: 10.1155/2022/2755246 (PMC9079324; doi:10.1155/2022/2755246)
Supplement: Supplementary 2 — Supplemental Table 1: the Preferred Reporting Items for Systematic Reviews and Meta-Analyses checklist. Supplemental Table 2: data extracted from enrolled studies concerning IL-1β in COVID-19 patients. Supplemental Table 3: data extracted from enrolled studies concerning IL-2 in COVID-19 patients and healthy controls. Supplemental Table 4: data extracted from enrolled studies concerning IL-4 in COVID-19 patients and healthy controls. Supplemental Table 5: data extracted from enrolled studies concerning IL-6 in COVID-19 patients and healthy controls. Supplemental Table 6: data extracted from enrolled studies concerning IL-8 in COVID-19 patients. Supplemental Table 7: data extracted from enrolled studies concerning IL-10 in COVID-19 patients and healthy controls. Supplemental Table 8: the Newcastle-Ottawa Scale (NOS) score showed the qualities of included studies. [file 2755246.f2.zip › Supplemental Table 7.docx]

**Supplemental Table 7.** Data extracted from enrolled studies concerning IL-10 in COVID-19 patients and healthy controls.

| Author (year) | country | Age (median /mean) | Time of sampling |  | CIOVID-19 patients | | | | | | | | | Healthy control(HC) | unit |
| --- | --- | --- | --- | --- | --- | --- | --- | --- | --- | --- | --- | --- | --- | --- | --- |
|  |  |  | **On hospital admission** | **Regular/ general/ ordinary** | **Mild/Moderate** | **non-severe/non-critical** | **Severe** | **Critical** | **Severe + Critical** | **non-survivor/died/death** | **Survivor/alive/survival** | **non-ICU** | **ICU** |  |  |
|  |  |  |  | n, mean (SD) or median (IQR) | n, mean (SD) or median (IQR) | n, mean (SD) or median (IQR) | n, mean (SD) or median (IQR) | n, mean (SD) or median (IQR) | n, mean (SD) or median (IQR) | n, mean (SD) or median (IQR) | n, mean (SD) or median (IQR) | n, mean (SD) or median (IQR) | n, mean (SD) or median (IQR) | n, mean (SD) or median (IQR) | pg/ml |
| Yuan XH(2020) | China | 66 (52, 69)，68 (61, 76)，67.5(57,85) | On hospital admission | 53, 4.58 (3.14, 5.05) |  |  |  |  | 54, 4.72 (3.55, 6.12) |  |  |  |  |  | pg/ml |
| McElvaney OJ（2020） | Ireland | 55.5(17.7) | On hospital admission |  |  |  |  |  |  |  |  | 20, 54.7 (15.7) | 20, 47.5 (17.4) |  | pg/ml |
| Lv ZH(2020) | China | 62 (23, 90) | On hospital admission |  | 115, 6.5 (4.84) |  | 155, 6.78 (6.1) | 84, 11.04(13.03) |  |  |  |  |  |  | pg/ml |
| Wu YJ(2020) | China | 61 (49, 69) | On hospital admission |  | 32, 2.34 (1.16, 4.41) |  | 39, 5.23 (3.31, 10.64) |  |  |  |  |  |  |  | pg/ml |
| Zou L(2020) | China | 65.0(55.0,71.5) | On hospital admission |  |  | 69, 6.11 (5.1, 7.07) | 52, 7.75 (6.15, 9.37) |  |  |  |  |  |  |  | pg/ml |
| Wang F(2020) | China | 68.6(9.0) | On hospital admission |  |  |  |  |  |  |  |  | 14, 5.2 (5, 7.5) | 14, 14.9 (5.9, 18.6) |  | pg/ml |
| Ke CJ(2020) | China | 62.55(14.18) | On hospital admission |  |  |  |  |  |  | 46, 8.69 (14.61) | 148, 5.06 (9.93) |  |  |  | pg/ml |
| Zhu Z(2020) | China | 50.90(15.26) | On hospital admission |  |  | 111, 3.13 (2.15, 4.57) | 16, 6.41 (3.24, 11.02) |  |  |  |  |  |  |  | pg/ml |
| Liu QQ(2020) | China | 55.0(39.0,67.0)，  66.0(55.5, 73.0)，70.0 (63.3, 78.8) | On hospital admission |  | 91, 5(5, 5.1) |  | 133, 5.9(5, 10.8) | 84, 10.9(6.4, 18.7) |  |  |  |  |  |  | pg/ml |
| Kwon JS (2020) | Korea | 50 (3.3) | On hospital admission |  | 6, 3.6(1.0, 5.1) (mild)  17,3.3(1.0,7.6) (moderate) |  |  |  | 8, 10(5.4, 36.1) |  |  |  |  |  | pg/ml |
| Liu SP(2020) | China | 64 (24, 92) | On hospital admission |  |  |  |  |  |  |  |  | 214, 5(5, 38.6) | 41, 10.1(5, 62.9) |  | pg/ml |
| Gadotti AC(2020) | Brazil | 61 (47, 73) | On hospital admission |  |  |  |  |  |  | 18, 293(226, 456) | 38, 318(217, 446) |  |  |  | pg/ml |
| Luo M(2020) | China | 61(49, 69) | On hospital admission |  |  |  |  |  |  | 201, 9.1(5.79, 16) | 817, 5.25(5, 6.8) |  |  |  | pg/ml |
| Zeng ZL(2020) | China | 62.0 (51.0, 70.0) | On hospital admission |  | 93, 5 (5, 6.8) |  | 167, 5.3 (5, 8.8) | 57, 9.5(6.7, 15.9) |  |  |  |  |  |  | pg/ml |
| Zhao Y(2020) | China | 48(37,63)(Patients)  48(40.75,52.25)(HC) | On hospital admission |  | 53, 3.68 (2.58, 6.18) |  | 18, 6.82 (3.36, 7.68) |  |  |  |  |  |  | 18, 3.92 (2.21, 6.02) | pg/ml |
| Han H(2020) | China | -  59.8(9.7)(HC) | On hospital admission |  | 42, 5.05 (4.59, 6.14) |  | 43, 5.3 (4.72, 6.02) | 17, 7.02 (5.3, 15.63) |  |  |  |  |  | 45, 3.91(3.29, 4.99) | pg/ml |
| Yi P(2020) | China | 54 (42, 64) | On hospital admission |  |  | 51, 3.45 (2.16, 5.82) | 49, 6.93 (4.35, 9.63) |  |  |  |  |  |  |  | pg/ml |
| Xu B(2020) | China | 62 (48.5, 71) | On hospital admission |  | 80, 5 (4.9, 7.16) |  | 45, 8.52 (4.9, 15.8) | 62, 9.56 (5.14, 14.8) |  |  |  |  |  |  | pg/ml |
| Dayarathna S(2020) |  |  | day 4–9 of illness |  | 15, 5.98(3.31, 11.57) |  | 8, 34.9(29, 65.46) |  |  |  |  |  |  |  | pg/ml |
| Dayarathna S(2020) |  |  | day 10–21of illness |  | 16, 5.98(5.98, 7.36) |  | 8,44.06(23.11,96.25) |  |  |  |  |  |  |  | pg/ml |
| Zhang BC(2020) | China | 62(47,78),  62.5(54.0, 69.0) | On hospital admission |  |  | 17,5.6(4.2,7.6) | 16,8(5.7,25) |  |  |  |  |  |  |  | pg/ml |
| Zhang BC(2020) | China | 49(37, 58),  48.0 (36, 57) | On hospital admission |  |  | 27,5.1(4.7,5.8) | 5,5.4(4.8,5.5) |  |  |  |  |  |  |  | pg/ml |
| Zhang BC(2020) | China | 66.5(56,73),  70.5 (63.0, 78.0) | On hospital admission |  |  | 13,5.4(4.4,6.6) | 34,9(7.3,14.4) |  |  |  |  |  |  |  | pg/ml |
| Zhang BC(2020) | China | 59.5 (54.5, 64),  62.0 (52.0, 74.5) | On hospital admission |  |  | 24,5.7(4.4,7.3) | 12,4.8(5.5,6.2) |  |  |  |  |  |  |  | pg/ml |
| Wan SX(2020) | China | 43.05(13.12),  61.29(15.55) | On hospital admission |  | 102,6.164(1.075) |  |  |  |  | 4,4.828(0.5765) | 17,4.452(0.381) |  |  |  | pg/ml |
| Tang YT(2020) | China | 59 (47, 68) | On hospital admission |  | 60,2.41(0.26) |  | 28,2.65(0.4) | 32,12.8(2.56) |  |  |  |  |  |  | pg/ml |
| Chen G(2020) | China | 56.0 (50.0, 65.0) | On hospital admission |  | 10,5(5,8.2) |  | 11,10.8(9.7,11.8) |  |  |  |  |  |  |  | pg/ml |
| Li XL(2020) | China | 44(32, 52),  56.5 (20, 72) | On hospital admission |  |  | 159,0(0,33) | 56,0(0,7.35) |  |  |  |  |  |  |  | pg/ml |
| Jin XH(2020) | China |  | On hospital admission |  |  | 105,3.52(0.19,22) | 40,4.37(1.54,39.5) |  |  |  |  |  |  |  | pg/ml |
| Hue S(2020) | France |  |  |  |  |  |  |  |  | 13,503.7(116.5) | 25,397.1(133.1) |  |  |  | pg/ml |
| Chen H(2020) | China | 63 (52, 70) | On hospital admission |  |  |  |  |  |  | 60,7(5,11.9) | 795,5(5,5) |  |  |  | pg/ml |
| Li CZ(2020) | China |  | On hospital admission |  |  | 754,5(5,5) |  | 210,7.5(5,12.6) |  |  |  |  |  |  | pg/ml |
| Li XJ(2020) | China | 43 (38, 47) | On hospital admission |  | 67,5(5,5) |  | 67,7.3(5,11.4) |  |  |  |  |  |  |  | pg/ml |

HC: healthy control.
