# Supplementary material for: Associations between Serum Interleukins (IL-1β, IL-2, IL-4, IL-6, IL-8, and IL-10) and Disease Severity of COVID-19: A Systematic Review and Meta-Analysis
Source: Biomed Res Int. 2022 Apr 30;2022:2755246. doi: 10.1155/2022/2755246 (PMC9079324; doi:10.1155/2022/2755246)
Supplement: Supplementary 2 — Supplemental Table 1: the Preferred Reporting Items for Systematic Reviews and Meta-Analyses checklist. Supplemental Table 2: data extracted from enrolled studies concerning IL-1β in COVID-19 patients. Supplemental Table 3: data extracted from enrolled studies concerning IL-2 in COVID-19 patients and healthy controls. Supplemental Table 4: data extracted from enrolled studies concerning IL-4 in COVID-19 patients and healthy controls. Supplemental Table 5: data extracted from enrolled studies concerning IL-6 in COVID-19 patients and healthy controls. Supplemental Table 6: data extracted from enrolled studies concerning IL-8 in COVID-19 patients. Supplemental Table 7: data extracted from enrolled studies concerning IL-10 in COVID-19 patients and healthy controls. Supplemental Table 8: the Newcastle-Ottawa Scale (NOS) score showed the qualities of included studies. [file 2755246.f2.zip › Supplemental Table 8.docx]

**Supplemental Table 8.** The Newcastle-Ottawa Scale (NOS) socre showed the qualities of included studies.

| Studies | SELECTION | | COMPARABILITY | | OUTCOME/EXPOSURE | | OVERALL |
| --- | --- | --- | --- | --- | --- | --- | --- |
| Xu X et al. |  | *** |  | * |  | ** | ****** |
| Kerget B et al. |  | ** |  | * |  | ** | ***** |
| Liu Q et al. |  | *** |  | ** |  | ** | ******* |
| Fan F et al. |  | ** |  | * |  | ** | ***** |
| Yuan XH et al. |  | *** |  | ** |  | ** | ******* |
| Sun Y et al. |  | *** |  | ** |  | ** | ******* |
| McElvaney OJ et al. |  | ** |  | * |  | ** | ***** |
| Zhang QH et al. |  | ** |  | * |  | ** | ***** |
| Li SH et al. |  | *** |  | ** |  | ** | ******* |
| Lv ZH et al. |  | **** |  | * |  | ** | ******* |
| Liu FF et al. |  | *** |  | * |  | ** | ****** |
| Wu YJ et al. |  | *** |  | * |  | ** | ****** |
| Yang F et al. |  | *** |  | * |  | ** | ****** |
| Zou L et al. |  | *** |  | * |  | ** | ****** |
| Wang F et al. |  | *** |  | * |  | ** | ****** |
| Li T et al. |  | **** |  | * |  | ** | ******* |
| Ke CJ et al. |  | *** |  | ** |  | ** | ******* |
| Shi PY et al. |  | *** |  | * |  | ** | ****** |
| Wang WL et al. |  | **** |  | ** |  | ** | ******** |
| Zhu Z et al. |  | *** |  | ** |  | ** | ******* |
| Liu Y et al. |  | ** |  | * |  | ** | ***** |
| Jurado A et al. |  | *** |  | ** |  | ** | ******* |
| Mandel M et al. |  | ** |  | * |  | * | **** |
| Liu QQ et al. |  | *** |  | ** |  | ** | ******* |
| Chen XH et al. |  | *** |  | ** |  | ** | ******* |
| Chen H et al. |  | **** |  | ** |  | ** | ******** |
| Kwon JS et al. |  | ** |  | ** |  | ** | ****** |
| Quartuccio L et al. |  | ** |  | * |  | * | **** |
| Liu SP et al. |  | *** |  | * |  | * | ***** |
| Gadotti AC et al. |  | ** |  | * |  | ** | ***** |
| Luo M et al. |  | *** |  | * |  | ** | ****** |
| Laguna-Goya R et al. |  | **** |  | ** |  | ** | ******** |
| Zeng ZL et al. |  | **** |  | ** |  | ** | ******** |
| Zhao Y et al. |  | *** |  | * |  | ** | ****** |
| Chen RC et al. |  | **** |  | * |  | ** | ******* |
| Li XJ et al. |  | **** |  | ** |  | ** | ******** |
| Huang HH et al. |  | ** |  | * |  | ** | ***** |
| Carlino MV et al. |  | ** |  | * |  | ** | ***** |
| Han H et al. |  | ** |  | * |  | ** | ***** |
| Yi P et al. |  | *** |  | * |  | ** | ****** |
| Xu B et al. |  | ** |  | * |  | ** | ***** |
| Effenberger M et al. |  | ** |  | * |  | ** | ***** |
| Maeda T et al. |  | *** |  | * |  | ** | ****** |
| Mikami T et al. |  | **** |  | * |  | * | ****** |
| Dayarathna S et al. |  | ** |  | * |  | * | **** |
| Zhang BC et al. |  | *** |  | * |  | ** | ****** |
| Dreher M et al. |  | ** |  | * |  | ** | ***** |
| Li CZ et al. |  | *** |  | ** |  | ** | ******* |
| Guirao JJ et al. |  | ** |  | * |  | ** | ***** |
| Wan SX et al. |  | ** |  | * |  | ** | ***** |
| Trecarichi EM et al. |  | ** |  | * |  | * | **** |
| Tang YT et al. |  | *** |  | * |  | ** | ****** |
| Chen G et al. |  | ** |  | * |  | ** | ***** |
| Chen JX et al. |  | **** |  | * |  | ** | ******* |
| Li XL et al. |  | *** |  | * |  | ** | ****** |
| Tu WJ et al. |  | * |  | * |  | ** | **** |
| Guo HQ et al. |  | ** |  | * |  | ** | ***** |
| Zhang L et al. |  | *** |  | * |  | ** | ****** |
| Jin XH et al. |  | *** |  | * |  | ** | ****** |
| Burian E et al.  Hue S et al. |  | **  ** |  | *  * |  | **  ** | *****  ***** |
